# Single-cell immune checkpoint landscape of PBMCs stimulated with *Candida albicans*

**DOI:** 10.1080/22221751.2021.1942228

**Published:** 2021-06-26

**Authors:** Weiwei Deng, Zhen Su, Panpan Liang, Yubo Ma, Yufang Liu, Kai Zhang, Yi Zhang, Tianyu Liang, Jin Shao, Xiao Liu, Wenling Han, Ruoyu Li

**Affiliations:** a Department of Dermatology and Venerology, Peking University First Hospital, Peking University; National Clinical Research Center for Skin and Immune Diseases; Beijing Key Laboratory of Molecular Diagnosis of Dermatoses, Beijing, People’s Republic of China; b Department of Dermatology and Venerology, The Third Affiliated Hospital of Sun Yat-Sen university, Guangzhou, People’s Republic of China; c Clinical laboratory, The Third Affiliated Hospital of Sun Yat-Sen university, Guangzhou, People’s Republic of China; d Department of Immunology, School of Basic Medical Sciences, Peking University Health Science Center, Peking University Center for Human Disease Genomics, Key Laboratory of Medical Immunology, Ministry of Health, Beijing, People’s Republic of China

**Keywords:** Single-cell RNA-sequencing, immune checkpoints, immunotherapy, *Candida albicans*, bioinformatics

## Abstract

Immune checkpoints play various important roles in tumour immunity, which usually contribute to T cells’ exhaustion, leading to immunosuppression in the tumour microenvironment. However, the roles of immune checkpoints in infectious diseases, especially fungal infection, remain elusive. Here, we reanalyzed a recent published single-cell RNA-sequencing (scRNA-seq) data of peripheral blood mononuclear cells (PBMCs) stimulated with *Candida albicans* (*C. albicans*), to explore the expression patterns of immune checkpoints after *C. albicans* bloodstream infection. We characterized the heterogeneous pathway activities among different immune cell subpopulations after *C. albicans* infection. The CTLA-4 pathway was up-regulated in stimulated CD4^+^ and CD8^+^ T cells, while the PD-1 pathway showed high activity in stimulated plasmacytoid dendritic cell (pDC) and monocytes. Importantly, we found that immunosuppressive checkpoints *HAVCR2* and *LAG3* were only expressed in stimulated NK and CD8^+^ T cells, respectively. Their viabilities were validated by flow cytometry. We also identified three overexpressed genes (*ISG20*, *LY6E*, *ISG15*) across all stimulated cells. Also, two monocyte-specific overexpressed genes (*SNX10*, *IDO1*) were screened out in this study. Together, these results supplemented the landscape of immune checkpoints in fungal infection, which may serve as potential therapeutic targets for *C. albicans* infection. Moreover, the genes with the most relevant for *C. albicans* infection were identified in this study.

## Introduction

*C. albicans* accounts for the most frequently isolated pathogen of candidemia [1], which causes approximately 40% mortality despite antifungal treatment [2]. Moreover, candidemia is a major challenge for healthcare as difficult diagnosis and therapy [3]. Fortunately, as technical development, single-cell RNA-sequencing (scRNA-seq) has been widely used to generate new scientific insights in various fields. Thus, efforts to analyze genomic transcripts of PBMCs stimulated with *C. albicans* based on single-cell levels will build a foundation for developing novel therapeutic targets and potential genes with the most relevant for *C. albicans* infection to improve the clinical outcomes of candidemia.

Immune checkpoints are inhibitory molecules of immune responses, which express in immune cells to avoid immune overreaction. These molecules can induce immune cells to an exhausted state. During the process, immune cells will reduce effector function and show an abnormal transcript in gene expression [4]. It is also clear that inhibitory interactions between immune checkpoints of immune cells are responsible for the immune evasion of pathogens and tumours [5]. Thus, immune checkpoint blockade treatment can overcome immune cell exhaustion and enhance the immune response during tumours and infections. In recent years, monoclonal antibodies (mAbs) targeting specific molecular components have been successfully prescribed to treat malignant tumours and autoimmune diseases [6–8]. For example, several pieces of evidence have shown that blocking immunosuppressive checkpoints (PD-1, CTLA-4, LAG-3, Tim-3/HAVCR2, and TIGIT) with mAbs would improve antitumor immune responses [9–12]. Although the functions of immune checkpoints in malaria, HIV, HBV, and tuberculosis have been roughly introduced[5], the transcripts of immune checkpoints between different immune cells during infections (especially during fungal and *Candida albicans* infections) are less well explored, let alone blocking them with mAbs for antifungal therapy. Thus, this move is vital as the detailed transcript profiles of immune checkpoints may provide novel strategies for treating infectious diseases.

In a recent study, de Vries et al. performed the scRNA-seq analyses for PBMCs stimulated with *C. albicans* [13]. They found that the deficiency of *LY86* would increase the susceptibility to candidemia by combining scRNA-seq with bulk transcriptomic analyses [13]. However, they did not explore heterogeneous pathway activities among immune cells or the transcripts of immune checkpoints. In this study, we reanalyzed the data to elucidate the single-cell expression patterns of immune checkpoint molecules, heterogeneous pathway activities and screen out the genes, which were the most relevant with *C. albicans* bloodstream infection. Importantly, we validated that the cell subpopulations would express immune checkpoints during *Candida albicans* infections using flow cytometry. Moreover, we also screened out the genes with the most relevant for *C. albicans* infection based on a weighted gene co-expression network analysis (WGCNA) algorithm.

## Materials and methods

### Ethical statement

This study was approved by the Clinical Research Ethics Committee of the Peking University First Hospital. We obtained blood samples from healthy donors and candidemia patients after obtaining informed consent.

### scRNA-seq and RNA-seq datasets

scRNA-seq data was downloaded from https://eqtlgen.org/candida.html. The data has been QC and cell type assignment by de Vries et al. [13], so we directly clustered cell subpopulations using their results. Bulk RNA-seq data was downloaded from the GEO database (GSE42606). PBMCs were stimulated with heat-killed *C. albicans* and RPMI control for 24 h in these data.

### Differential expression genes and gene set enrichment analysis of immune cells

scRNA-seq data were clustered by the Seurat package (version 3.1.5). Then, t-SNE was used to visualize the single cells (A total of 15,085 cells, included 7,160 stimulated cells and 7,925 unstimulated cells). Differential expression genes (DEGs, adjusted *p*-value < 0.05, |log_2_ fold change| >0.5) were identified by FindMarkers function in the Seurat package. Finally, 88, 112, 435, 331, 104, 84 DEGs were identified in B cells, CD4^+^ T cells, CD8^+^ T cells, monocytes, NK cells, and pDC cells, respectively. The volcano plot of DEGs was generated by ggplot2 (version 3.3.2), ggrepel (version 0.8.2) package, and dplyr (version 1.0.0) package. Gene set enrichment analysis (GSEA) was conducted using ClusterProfiler (version 3.14.3) package. Venn diagram was drawn using the online database (http://jvenn.toulouse.inra.fr/app/example.html).

### Heterogeneous pathway activity analyses

Gene ontology (GO) analysis was carried out using a free online website (Metascape: http://metascape.org). The scores of hallmark pathways were calculated using a new algorithm [14]. The algorithm can evaluate the relative regulation of pathways by comparing different samples. The gene sets of hallmark pathways were downloaded from the GSEA database (https://www.gsea-msigdb.org/gsea/index.jsp).

### Flow cytometry of PBMCs

PBMCs were isolated from the whole blood of 3 healthy donors as previous methods which was described by de Vries et al [13]. Heat-killed *C. albicans* blastoconidia (strain SC5314, 1 × 10^6^ CFU/ml) was used to infect PBMCs (5 × 10^5^ cells) [13]. After 24 h, cells were washed twice in phosphate-buffered saline (PBS). Cell suspensions were then incubated with anti-human Fixable Viability Dye eFluor™ 506 (live, 65-0866-14, Thermo Scientific), CD45 (368524; BioLegend), CD3(300408; BioLegend), CD8A (300911; BioLegend), CD56 (362546; BioLegend), Tim-3 (345016; BioLegend), LAG-3 (369314; BioLegend) for 30 min (1:300) in PBS. The stained cells were then washed twice. Finally, the stained cells were analyzed on the flow cytometer (BD Biosciences). FlowJo software (version 10.4.0) was used to analyze the data.

### WGCNA analysis

We isolated the normalized expression matrixes of monocytes, CD8^+^ T cells, and NK cells from Seurat’s object using the subset algorithm in R. Then, WGCNA’s (version 1.69) standard process was conducted [15]. During the process, all genes were analyzed to screen out the novel biomarkers, which significantly correlated with the trait stimulated with *C. albicans*. In brief, we first calculated Softpower values, Softpower 4 was selected to construct the modules of all cells. Then, TOM dissimilarity was used to identify the modules of all cells. There was a minimum size of 50 genes in each module. We also chose other values as the minimum size of the gene, for example, 20 and 30, which emerged so many interfered modules. Thus, we chose 50 as the minimum size of the gene group in each module according to the published literatures [16,17]. Subsequently, the correlations between modules and the traits (PBMCs were stimulated with *C. albicans* or unstimulated with RPMI) were identified by detecting the correlations between module eigengene and the traits. After that, scatter plots of module eigengenes in the models were generated to identify the correlations of module membership (MM) and gene significance (GS). Therefore, the modules with the highest R values between modules and the traits, and the first rank relationships between MM and GS, were considered as the most relevant modules. Finally, hub genes, mostly related to the stimulated trait, were screened out by ranked top 10 degrees. These genes were visualized at the single-cell level using t-SNE.

### The bulk RNA-seq analysis

We first normalized this data using TPM. Then, a matrix was generated using log_2_(TPM+1). The transcript levels of immune checkpoints, the genes with the most relevant for *C. albicans* infection were identified in bulk RNA-seq using ggstatsplot (version 0.5.0) package.

### Real-time reverse transcriptase-polymerase chain reaction (RT–PCR) validated gene expression

RT–PCR was conducted to validate the expression of *ISG20*, *LY6E*, *ISG15*, *SNX10*, and *IDO1*. PBMCs of three healthy donors were stimulated with Heat-killed *C. albicans* for 24 h. Then, cells were lysed using TRIZOL to extract RNA. Also, the whole blood samples from three Candidemia patients and three healthy donors were collected to extract RNA. Complementary DNA was obtained using primer Script™ RT reagent Kit with gDNA eraser (TaKaRa). Then RT–PCR was conducted in triplicate according to the manual of PowerUp SYBR Green Master Mix (life). The relative gene expression of genes was calculated using 2^−ΔΔCt^method. The primers for RT–PCR were given in table S1.

### Statistical analysis

We conducted the statistical analyses for DEGs, GO, GSEA, WGCNA in the default build-in methods of respective packages or online databases [18]. The statistical analysis of pathway activities was conducted using the random permutation test. Paired t-test was used to analyze the results of flow cytometry. The bulk RNA-seq expression was statistically analyzed using the ggstatsplot package based on a student’s t-test. An unpaired t-test was used to analyze the results of RT–PCR.

## Results

### Monocytes and pDC cells up-regulated MAPK and toll-like receptor signalling during *C. albicans* infection

**Figure 1.**
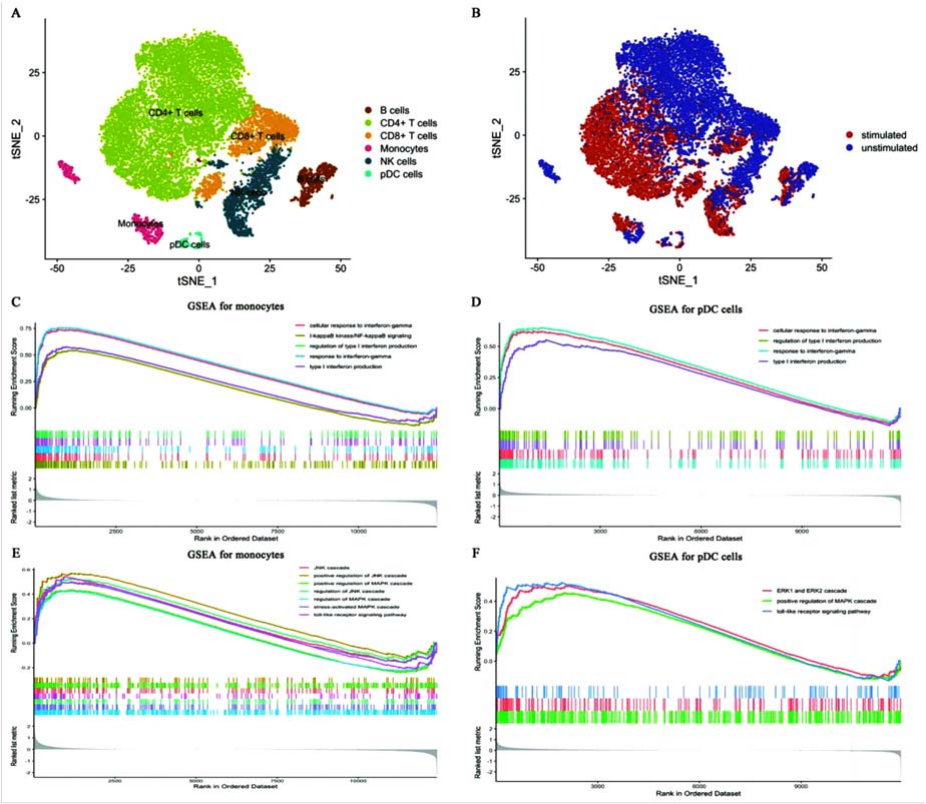
**Immune cell subpopulations and GSEA analysis** (A-B) The t-SNE projection of all cells. (A) All cell subpopulations. Different colours represent different cell subpopulations, dots represent cells. (B) The detailed treatment of PBMCs. (C-D) GSEA analysis for monocytes (C), pDC cells (D). (E-F) GSEA identified up-regulated antifungal pathways for monocytes (E) and pDC cells. Running Enrichment score represents pathway activity. If it is beyond 0 represents up-regulated activity, otherwise, it represents down-regulated pathway activity.

The t-SNE scatter plot of single-cell PBMCs was shown in Figure 1A. CD4^+^ T cells, CD8^+^ T cells, NK cells, B cells, monocytes, and pDC cells existed in stimulated or unstimulated PBMCs (Figure 1A). Among these cells, CD4^+^ T cells made up the most amount with a straightforward look (Figure 1A). The detailed treatment of PBMCs was shown in Figure 1B, which showed the distribution of stimulated or unstimulated PBMCs.

After clustering cell subpopulations, we conducted gene set enrichment analysis (GSEA), which can identify the regulations of pathways based on all genes, rather than differential expression genes (DEGs). We observed that interferon-gamma and type I interferon signalling were up-regulated across all stimulated cells (Figure 1C-D, Figure S1), indicating their important roles in fungal immunity (especially identifying their regulation across different cell subpopulations). Multiple observational studies have recently demonstrated that interferon-gamma and type I interferon signalling played indispensable roles in the clearance of *Aspergillus fumigatus* [19,20]. However, I-kappaB kinase/ NF-kappaB signalling showed an increased activity only in CD4^+^ T cells, CD8^+^ T cells, NK cells, B cells, and monocytes (Figure 1C, Figure 1D, Figure S1). Notably, these pathways were all up-regulated in stimulated CD8^+^ T cells, which were given scant attention during past antifungal researches, indicating the cell with a potential antifungal property. Also, we observed that MAPK and toll-like receptor signalling were only activated in stimulated monocytes and pDC cells (Figure 1E, Figure 1F). Several previous evidences have identified that MAPK and toll-like receptor signalling orchestrated innate immune responses against *C. albicans* infection [21–25]. Together, these results highlighted that monocytes and pDC cells played important roles in innate immune, while other cells may only activate interferon-related pathways to achieve antifungal responses.

### The functional analyses of DEGs

**Figure 2.**
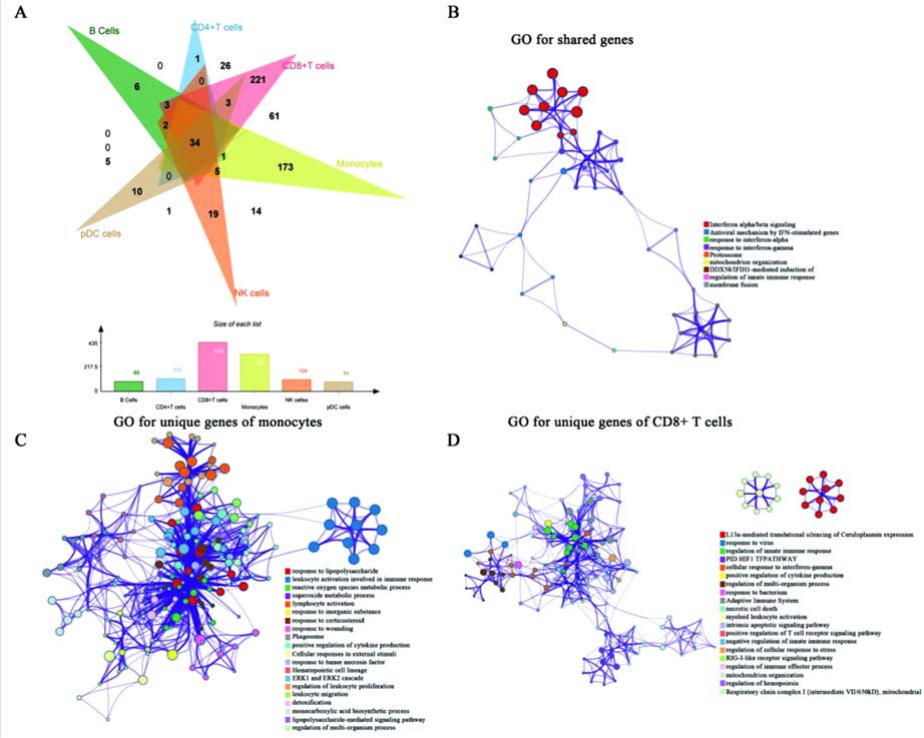
**The results of DEGs and GO analysis** (A) Venn diagram for DEGs of all cell subpopulations. The bar diagram showed the numbers of DEGs among different immune cells. (B-D) GO analysis was conducted for shared DEGs (B), unique DEGs of monocytes (C), and unique DEGs of CD8^+^ T cells (D). Nodes represented the enriched terms. Size of nodes corresponding to significant levels. The thickness of the line represented the numbers of shared genes between terms.

DEGs were identified by comparing stimulated cells with unstimulated cells in the same cell subpopulation (Figure 2A, Figure S2). Among these DEGs, interferon-related genes were overexpressed in all cells (genes with adjusted *p*-value < 0.05, log_2_ fold change >1) (Figure S2). Chemokine CXCL10, which can inhibit the growth of *C. albicans* in vitro and accelerate fungal clearance in animal models [26], was overexpressed in CD4^+^ T cells, B cells, and monocytes (Figure S2). There remained many other overexpressed genes (Figure S2) in the stimulated cells. These genes may provide important clues for subsequent studies. Venn diagram (Figure 2A) showed that 1, 221, 173, 19, 10, and 6 unique DEGs were founded in CD4^+^ T cells, CD8^+^ T cells, monocytes, NK cells, pDC cells, and B cells, respectively. The unique DEGs may reflect specific responses to *C. albicans* infection. Strikingly, CD8^+^ T cells with the maximum number of unique DEGs highlighted important antifungal immunity roles (Figure 2A). We also observed that 34 DEGs were existed in all cell subpopulations (Figure 2A), suggesting coordinated responses against *C. albicans* infection among different cells.

After identifying DEGs, we analyzed the shared and unique DEGs using GO analysis to explore the potential functions of these genes. In line with GSEA analysis, interferon-related pathways were significantly enriched in all stimulated cells (Figure 2B). Other new functions such as the proteasome, mitochondrion organization, and regulation of innate immune response were significantly enriched in all cells (Figure 2B). Metabolic functions and immune-related functions, especially for antifungal immunity, such as reactive oxygen species metabolic process, ERK1/2 cascade, and leukocyte proliferation and migration were only significantly enriched in monocytes, revealing the specific antifungal functions of monocytes (Figure 2C). Importantly, antipathogen functions such as response to virus and bacterium were significantly enriched in CD8^+^ T cells (Figure 2D). Moreover, the adaptive immune system and the regulation of innate immune response were also significantly enriched in CD8^+^ T cells (Figure 2D). In contrast, chemokine receptors binding chemokines, p53 signalling pathway, and platelet degranulation were mainly enriched in NK cells (Figure S3A). There were only two functions (neutrophil degranulation and cellular response to hormone stimulus) that were enriched in pDC cells (Figure S3B). However, unique DEGs of CD4^+^ T cells and B cells were not enriched in any functions. The above findings collectively suggested that CD8^+^ T cells, monocytes, and NK cells may be the main effector cells to fight against early *C. albicans* infection.

### Immune checkpoint pathways were activated during *C. albicans* infection

**Figure 3.**
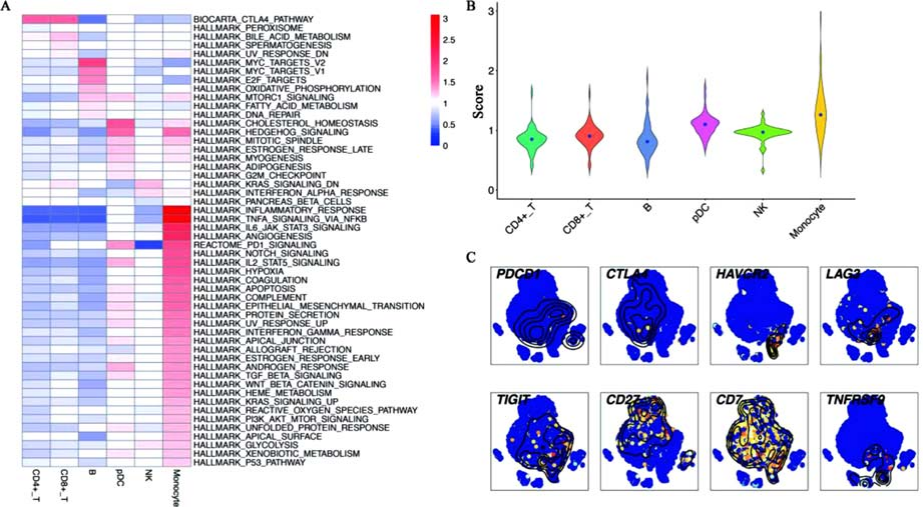
**Heterogenous pathway activities and the transcript levels of immune checkpoints (A)** The heatmap for pathway activities of all cell subpopulations. Red represented high viability and blue represented low activity. Monocytes up-regulated several immune pathways during infection. (B) The violin diagram showed the average score of pathways among all immune cells. Monocytes showed the highest pathway activity. (C) t-SNE identified the single-cell expressions of immune checkpoints and costimulatory molecules. High expression was in yellow and red nodes, while low expression was drawn in blue.

Given the specific functions of different cell subpopulations during *C. albicans* infection, we employed hallmark gene sets, PD-1, and CTLA-4 signalling pathways to analyze the heterogeneity of pathway activity among stimulated cell subpopulations (Figure 3A, Figure 3B). This analysis was to score all genes, which existed in a certain pathway including DEGs and non-DEGs. Notably, we observed that pathway activities showed obvious heterogeneities among different cell subpopulations stimulated with *C. albicans* (Figure 3A). The highest pathway activity was found in monocytes, followed by pDC cells, NK cells, CD8^+^ T cells, CD4^+^ T cells, and B cells (Figure 3A, Figure 3B). Immune-related activity such as inflammatory response, TNF signalling via NFKB, IL6 JAK STAT3 signalling, and PI3K AKT MTOR signalling et al. ranked first in monocytes (Figure 3A), indicating that maintaining high viability of these pathways may be important for *C. albicans* clearance. Strikingly, the CTLA-4 pathway was up-regulated in both CD4^+^ and CD8^+^ T cells, while the high activity of PD-1 signalling was observed in monocytes and pDC cells (Figure 3A). It is well known that CTLA-4 and PD-1 signalling inhibit the immune activity of immune cells. Hence, the high viability of these pathways may cause immunosuppression of antifungal functions and restrict pathogens’ clearance. In summary, these results identified the heterogeneous pathway activity that existed in stimulated cell subpopulations. Most importantly, the new findings advance our understanding of immunosuppressive checkpoint pathways in *C. albicans* infection.

### Immunosuppressive checkpoints *HAVCR2* and *LAG3* specifically overexpressed in NK and CD8^+^ T cells, respectively

**Figure 4.**
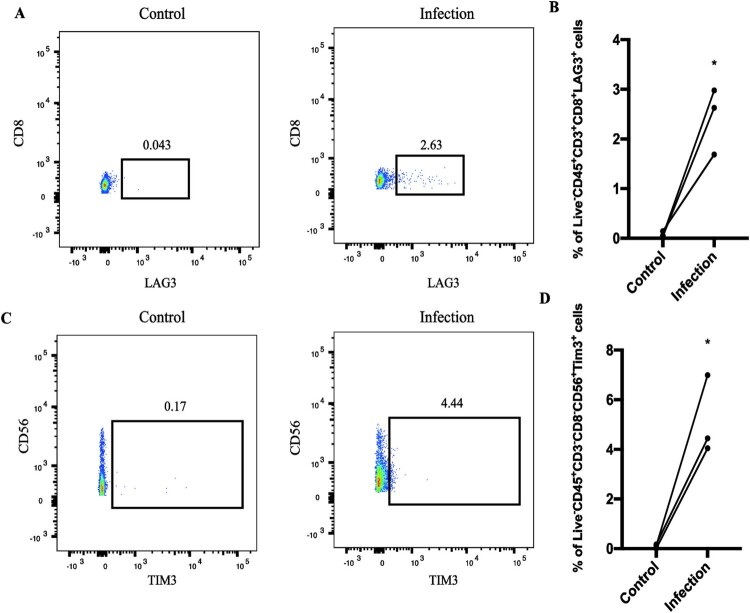
***LAG3* and *HAVCR2* were highly expressed during *C. albicans* infection.** After infection, PBMCs were analyzed by flow cytometry. (A) The percent of live CD45^+^CD3^+^CD8^+^LAG3^+^. (B) Statistical analysis of live CD45^+^CD3^+^CD8^+^LAG3^+^ from 3 healthy donors. (C-D) The flow cytometry results of *HAVCR2* based on 3 healthy donors. (*p < 0.05; **p < 0.01; ***p < 0.001). CD45^+^CD3^+^CD8^+^LAG3^+^ and CD45^+^CD3^+^CD8^+^LAG3^+^ increased cell numbers after *C. albicans* infection.

Because CTLA-4 and PD-1 pathways were up-regulated in stimulated cells, we formulated a hypothesis that immunosuppressive checkpoints would also show high transcript levels in certain cell subpopulations after *C. albicans* infection. In agreement with the hypothesis, we observed that *HAVCR2* and *LAG3* only expressed in stimulated NK and CD8^+^ T cells, respectively (Figure 3C, Figure 1A, Figure 1B). However, *PDCD1*, *CTLA4*, and *TIGIT* were lowly expressed across all cells and showed no specific expression in a certain cell subpopulation (Figure 3C, Figure 1A, Figure 1B). Strangely, among costimulatory molecules (*CD27*, *TNFRSF9*), which enhance T cell effector functions [27,28], *CD27* was down-regulated in stimulated CD4^+^ T cells and B cells (Figure 3C, Figure 1A, Figure 1B). However, *TNFRSF9* was up-regulated in stimulated pDC cells. This may reflect that the effector functions of CD4^+^ T cells and B cells were dampened during *C. albicans* infection. *CD7* is another costimulatory molecule, whose normal expression is limited to T and NK cells [29]. We did not observe a distinct expression between before and after stimulation (Figure 3C, Figure 1A, Figure 1B). We then performed flow cytometry to validate the viabilities of *HAVCR2* and *LAG3* after *C. albicans* infection. We observed that the number of CD45^+^CD3^+^CD8^+^LAG3^+^ (Figure 4A, 4B) and CD45^+^CD3^−^CD8^−^CD56^+^Tim3^+^ cells (Figure 4C, 4D) would be significantly improved after *C. albicans* infection (Figure S4 showed the gating strategy during flow cytometric analysis). In line with the above findings, the transcript levels of these molecules showed the same trend in the bulk RNA-seq levels (Figure S5A). Thus, RNA-seq analysis also proved that the results of these molecules’ expression in single-cell levels were reliable. The expression patterns of these immune checkpoints and costimulatory molecules may reflect the heterogenous immune responses for *C. albicans* bloodstream infection among various immune cells. High viabilities of *HAVCR2* and *LAG3* may limit the immune effectors of NK and CD8^+^ T cells during *C. albicans* infection, which leads to eliminating pathogens inefficiently. However, other immune checkpoints (*PDCD1*, *CTLA4*, and *TIGIT*) may not involve in the immune regulations during *C. albicans* infection. Low expression of *CD27* in infected CD4^+^ T cells and B cells indicates that the host may inhibit excessive immune responses by limiting the viability of *CD27*. *TNFRSF9* is only expressed in stimulated pDC cells, which may reveal that a specific immune response will be initiated to combat *C. albicans*.

**Figure 5.**
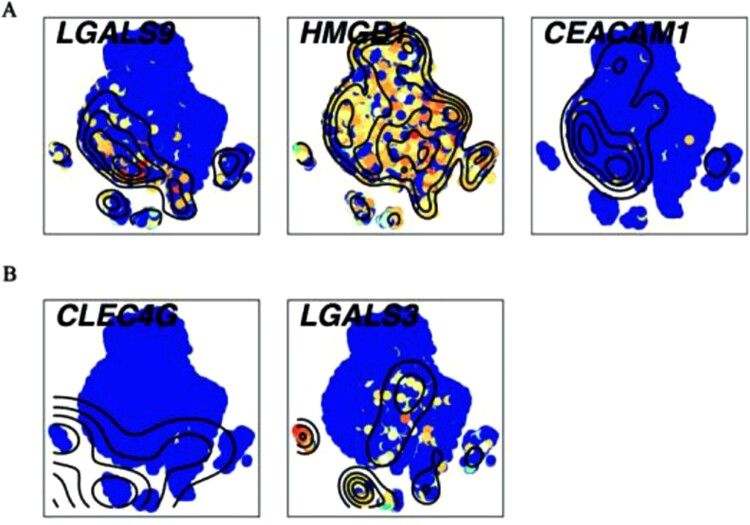
**Single-cell transcript levels of immune checkpoint’s ligands (A)** The ligands for Tim-3/HAVCR2. (B) The ligands for LAG-3. High expression was in yellow and red nodes, while low expression was drawn in blue.

Ligands combined with Tim-3/HAVCR2 are Galectin-9 (*LGALS9*) [30], HMGB-1(*HMGB1*) [31], and CEACAM-1 (*CEACAM1*) [32]. There are several ligands for LAG-3 including LSECtin (*CLEC4G*), galectin-3 (*LGALS3*), and MHC class II molecules [33]. To identify which cells influenced Tim-3/HAVCR2 and LAG-3, we explored the expression of these ligands except MHC class II molecules, which included several unclear ligands of LAG-3. We observed that *HMGB1* was expressed across all cells, suggesting that all cells may interact with Tim-3/HAVCR2 (Figure 5A). However, *LGALS9* was specifically expressed in stimulated CD4^+^ T cells, CD 8^+^ T cells, NK cells, B cells, monocytes, and unstimulated pDC cells (Figure 5A). Strangely, we did not find the expression of *CEACAM1*(Figure 5A). The expressions of LAG-3 ligands showed that *LGALS3* expressed in all monocytes and stimulated pDC cells, while *CLEC4G* did not express in any cells (Figure 5B). The results of bulk RNA-seq revealed that *LGALS9*, *CEACAM1*, and *LGALS3* showed relatively higher viabilities in stimulated samples compared with unstimulated controls (Figure S5B). It was possible that subtle changes might have been enlarged in the bulk levels. These new findings of Tim-3/HAVCR2, LAG-3, and their ligands shed light on the important roles of immunosuppressive checkpoints in *C. albicans* infection.

### WGCNA screened out the hub genes

**Figure 6.**
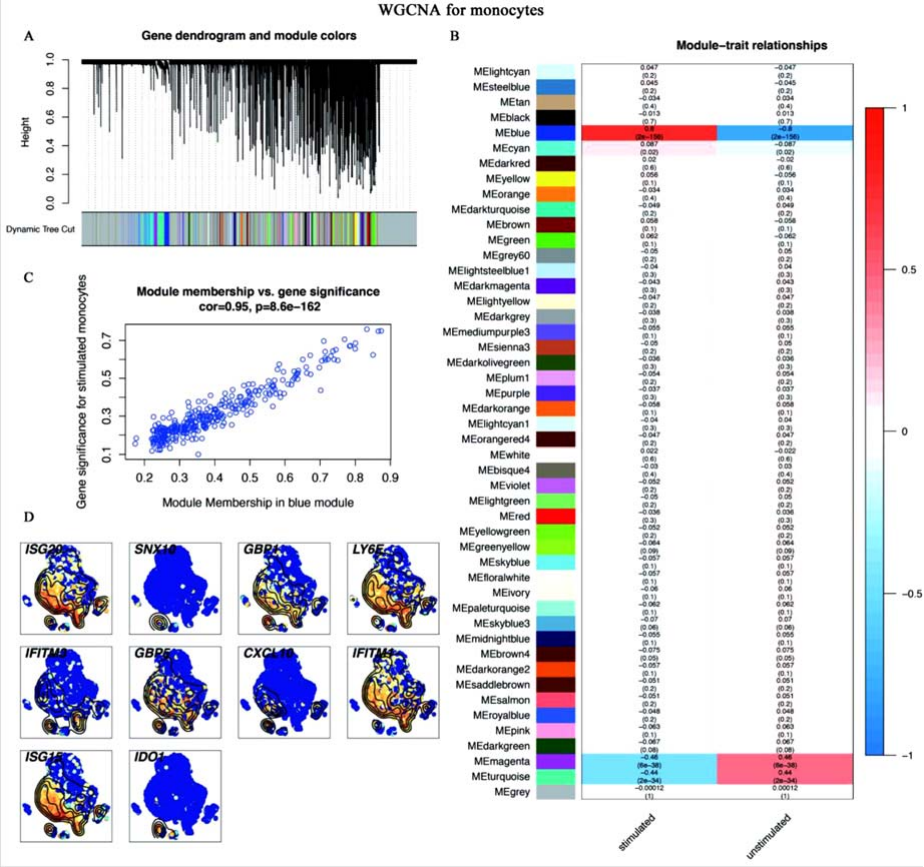
**WGCNA for monocytes** (A-C) The results of WGCNA. (A) Gene dendrogram and module colours. The genes with similar expression patterns were divided into the same modules. (B) The relationships between all modules and traits. Red represented positive correlations and blue represented negative correlations. The blue module was identified in the subsequent analyses. (C) Scatter plot of module eigengenes in the blue module. This represented the correlations between genes and the blue module. (D) The single-cell transcript levels of top 10 hub genes.

**Figure 7.**
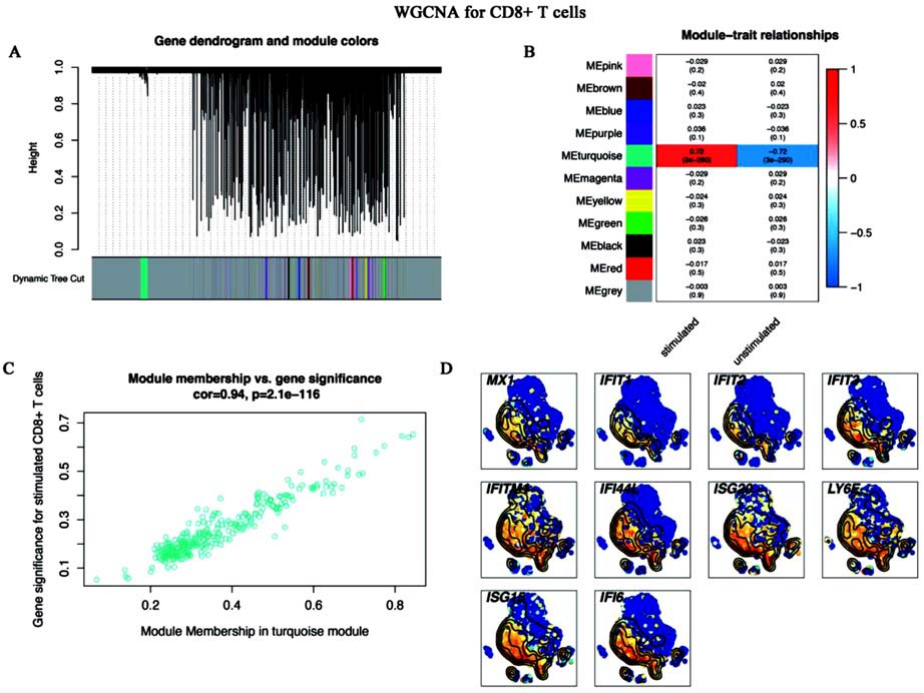
**WGCNA for CD8^+^ T cells** (A-C) WGCNA screened out the hub genes of CD8^+^ T cells. (A) Gene dendrogram and module colours. The genes with similar expression patterns were divided into the same modules. (B) The relationships between all modules and traits. Red represented positive correlations and blue represented negative correlations. The turquoise module was identified in the subsequent analyses. (C) Scatter plot of module eigengenes in the turquoise module. This represented the correlations between genes and the turquoise module. (D) The single-cell transcript levels of top 10 hub genes.

**Figure 8.**
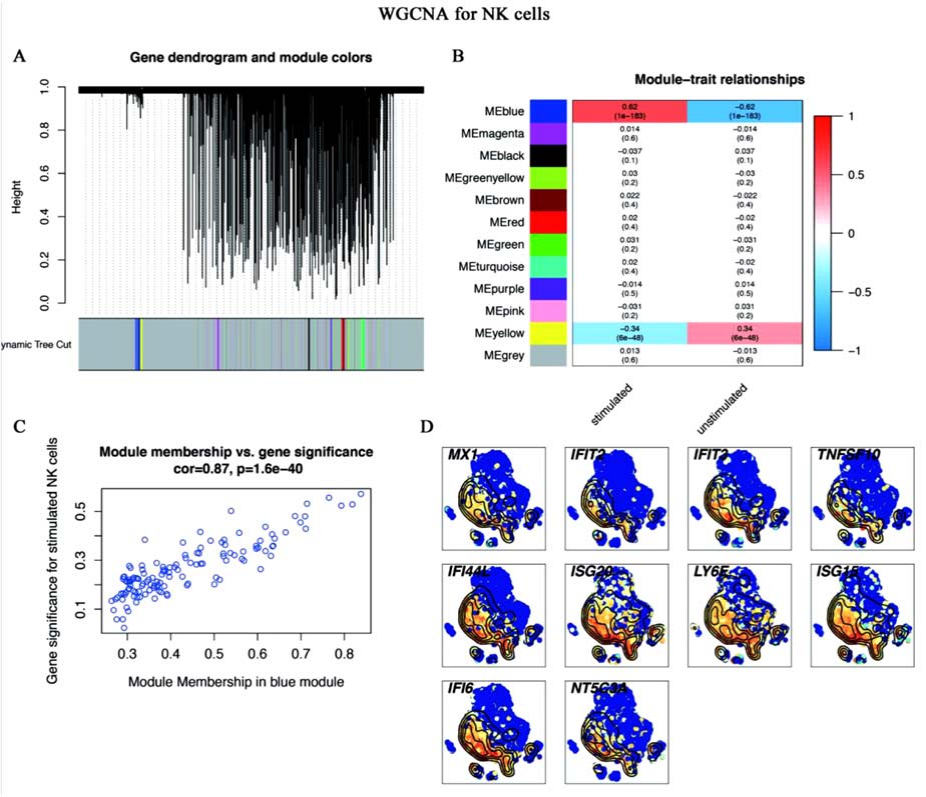
**WGCNA for NK cells** (A-C) WGCNA algorithm was used to construct co-expression modules. (A) Gene dendrogram and module colours. The genes with similar expression patterns were divided into the same modules. (B) The relationships between all modules and traits. The blue module was identified in the subsequent analyses. Colours corresponding to the degree of correlations between clinical traits and modules. (C) Scatter plot of module eigengenes in the blue module. This represented the correlations between genes and the blue module. (D) The single-cell transcript levels of top 10 hub genes.

Given the specific functions of DEGs in monocytes, CD8^+^ T cells, and NK cells (Figure 2), we conducted WGCNA to screen out these cells’ hub genes related to the trait of *C. albicans* infection. After WGCNA for monocytes, 48 modules were generated (Figure 6A). Then, the blue module was identified as the key module (R=0.8, *p*<0.0001) (Figure 6B), and the correlation between module membership (MM) and gene significance (GS) in blue also confirmed that (Figure 6C). The hub genes of the blue modules were screened out by ranking top 10 degrees. Finally, 10 genes were identified as the hub genes, which were the most relevant to the trait of *C. albicans* infection in stimulated monocytes (Figure 6D). After the same pipeline, a turquoise module (Figure 7A, Figure 7B, Figure 7C) and a blue module (Figure 8A, Figure 8B, Figure 8C) were identified as the key modules of CD8^+^ T and NK cells, respectively. Figure 7D and Figure 8D illustrated the top 10 hub genes of CD8^+^ T and NK cells, respectively.

### *ISG20*, *LY6E*, and *ISG15* could be the genes with most relevant for *C. albicans* infection and *SNX10* and *IDO1* may serve as antifungal functions

After identifying hub genes, we intersected these genes among different cells (Figure S6A). *ISG20*, *LY6E,* and *ISG15* were the hub genes of the three cell subpopulations. These genes were overexpressed across all cells after *C. albicans* infection (Figure S2, Figure 6D), except *LY6E* had no overexpression in NK cells (Figure S2C). Importantly, the high transcript levels of *ISG20*, *LY6E,* and *ISG15* were also observed in the stimulated bulk samples (Figure S6B). Among monocytes’ hub genes, we noticed that *SNX10* and *IDO1* were only overexpressed in stimulated monocytes (Figure 6D, Figure S2E). Also, *SNX10* and *IDO1* showed high vitalities in the stimulated bulk samples (Figure S6B). Importantly, we validated the transcript levels of these genes in PBMCs (Figure S7A) and the whole blood of candidemia patients (Figure S7B) using RT–PCR. A previous study reported that *SNX10* deficiency would decrease *Listeria monocytogenes* clearance of macrophages, and SNX10-deficient mice were more susceptible to the bacteria [34]. *IDO1* has been demonstrated that deficiency of this gene was associated with vulvovaginal candidiasis (VVC) and risk factors for recurrent VVC [35]. Based on our data and previous studies, we could conclude that *SNX10* and *IDO1* may serve as the novel antifungal genes needed for further research. Besides, we also screened out the overexpressed genes with the most relevant for *C. albicans* infection.

### Discussion

Because the traditional antifungal pharmacotherapy is suboptimal for immunocompromised patients, immune enhancement strategies were urgent to strengthen the therapeutic effect of antifungal agents [36]. Immune checkpoint inhibitor therapy, a rapidly emerging immunotherapeutic programme that has been used in the effective treatment of multiple cancer types [37]. This immunotherapy strategy can release the restriction of immunosuppressive checkpoints to enhance the anti-tumour immune effects [37]. Moreover, immune checkpoint inhibitor therapy is acquiring more attention to infectious diseases [38]. Indeed, Wurster et al. firstly reported that blockade of the PD-1 in vivo (animal model) would contribute to a survival advantage and accelerate *Aspergillus fumigatus* clearance in lung [39]. Also, an in vitro study revealed that target PD-L1 would promote a protective immunity to *Aspergillus fumigatus* [40]. Hence, strategies to employ checkpoint blockade may be useful for the immune enhancement of fungal infected patients. Until then, understanding the expression of immune checkpoints during fungal infection can help establish the strategy and optimize treatment windows for immunotherapies.

Here, we first explored the heterogenous pathway activities of different cell subpopulations after *C. albicans* infection. Consistent with previous studies, monocytes with activating antifungal pathways (Figure 1C, Figure 1E, Figure 2C, Figure 3A-B) were the major cell subpopulations that combat *C. albicans* [41–44]. Notably, CD8^+^ T cells, which remain less well known in antifungal immunity, showed immune-related pathways’ activities during *C. albicans* stimulation (Figure S1B, Figure 2D). Also, some findings have highlighted that CD8^+^ T cells mediated antifungal protection in the depletion of CD4^+^T and B cells [45,46]. Combined with these studies, we could have insight into the crucial roles of CD8^+^ T cells in antifungal immunity, which needed to be further investigated in this field. We also observed that *HAVCR2* and *LAG3* showed high viabilities in stimulated NK and CD8^+^ T cells (Figure 4), respectively. A published study found that Tim-3/HAVCR2 could negatively regulate NK cell-mediated cytotoxicity [47]. Similarly, LAG-3 exerts differential immunosuppressive impacts on several types of lymphocytes by inhibiting cell ability and the secretion of cytokines. Thus it may be a promising therapy target [48,49]. Importantly, *HMGB1* (a ligand for Tim-3/HAVCR2) expressed across all cells (Figure 5A), indicating that various types of immune cells may restrain the effector activity of NK cells. In comparison, the ligand (*LGALS3*) for LAG-3 had the only limited expression in all monocytes and stimulated pDC cells, thus, these cells may also negatively regulate the antifungal immunity of CD8^+^ T cells. In addition, we also screened out several overexpressed genes, which were most relevant with candidemia, and two promising antifungal genes using WGCNA.

Collectively, this study characterized the heterogeneous pathway activities of different immune cells during *C. albicans* infection based on single-cell levels. Also, the functional analyses of CD8^+^ T cells during *C. albicans* infection may be acquired in our study. Moreover, comprehensive analyses and experimental validations of immune checkpoints will strengthen the notion that immune checkpoint inhibitor therapy may be a new antifungal immunity strategy. Specifically, the promising genes with the most relevant for *C. albicans* infection could be employed to conduct antifungal researches to ameliorate the outcomes. However, the detailed mechanisms of the above findings are still needed to explore by proper experimental designs. Thus, we will conduct further researches.

## Supplementary Material

Supplementary_figures.zip

Table_S1.xlsx
